# Betaine Promotes Fat Accumulation and Reduces Injury in Landes Goose Hepatocytes by Regulating Multiple Lipid Metabolism Pathways

**DOI:** 10.3390/ani12121530

**Published:** 2022-06-13

**Authors:** Jiying Liu, Ruilong Song, Shengyan Su, Nannan Qi, Qifa Li, Zhuang Xie, Shali Yu

**Affiliations:** 1 School of Biotechnology, Jiangsu University of Science and Technology, Zhenjiang 212018, China; liujiying@just.edu.cn (J.L.); qinannan@stu.just.edu.cn (N.Q.); 2 College of Veterinary Medicine, Yangzhou University, Yangzhou 225009, China; rlsong@yzu.edu.cn; 3 Key Laboratory of Freshwater Fisheries and Germplasm Resources Utilization, Ministry of Agriculture, Freshwater Fisheries Research Center, Chinese Academy of Fishery Sciences, Wuxi 214081, China; susy@ffrc.cn; 4 College of Animal Science and Technology, Nanjing Agricultural University, Nanjing 210095, China; liqifa@njau.edu.cn (Q.L.); zxie@njau.edu.cn (Z.X.); 5 Nantong Key Laboratory of Environmental Toxicology, Department of Occupational Medicine and Environmental Toxicology, School of Public Health, Nantong University, Nantong 226019, China

**Keywords:** betaine, Landes goose, fatty liver, fat metabolism, lipid droplets

## Abstract

**Simple Summary:**

Betaine is a safe, effective, naturally occurring trimethylglycine, which is widely used as a nutritional supplement in animal husbandry. Early research showed betaine redistributes body fat by regulating systemic fatty acid metabolism. We previously found that betaine reduces sebum thickness and abdominal fat, and increases liver weight and triglyceride content, in Landes geese fed with high-energy carbohydrates. However, the mechanism underlying these effects remained unclear. Here, we present an in vitro Landes goose fatty liver model, which was developed using primary hepatocytes and a high-glucose medium simulating the conditions of high-energy carbohydrate feeding in vivo. We used this model to investigate the effects of betaine supplementation. Our results suggest that betaine increases lipid deposition, reduces lipid droplet size, and restores mitochondrial membrane potential (β-oxidation) and the expression of genes involved in lipid hydrolysis transfer genes. By contrast, the expression of fatty acid synthesis genes was down-regulated. The comprehensive effect led to an increase in total triglyceride in the liver, but a healthier steatosis phenotype. Overall, these results show that betaine could improve the quality of foie-gras while decreasing the injury to geese that results from overfeeding.

**Abstract:**

Betaine is a well-established supplement used in livestock feeding. In our previous study, betaine was shown to result in the redistribution of body fat, a healthier steatosis phenotype, and an increased liver weight and triglyceride storage of the Landes goose liver, which is used for foie-gras production. However, these effects are not found in other species and strains, and the underlying mechanism is unclear. Here, we studied the underpinning molecular mechanisms by developing an in vitro fatty liver cell model using primary Landes goose hepatocytes and a high-glucose culture medium. Oil red-O staining, a mitochondrial membrane potential assay, and a qRT-PCR were used to quantify lipid droplet characteristics, mitochondrial β-oxidation, and fatty acid metabolism-related gene expression, respectively. Our in vitro model successfully simulated steatosis caused by overfeeding. Betaine supplementation resulted in small, well-distributed lipid droplets, consistent with previous experiments in vivo. In addition, mitochondrial membrane potential was restored, and gene expression of fatty acid synthesis genes (e.g., sterol regulatory-element binding protein, diacylglycerol acyltransferase 1 and 2) was lower after betaine supplementation. By contrast, the expression of lipid hydrolysis transfer genes (mitochondrial transfer protein and lipoprotein lipase) was higher. Overall, the results provide a scientific basis and theoretical support for the use of betaine in animal production.

## 1. Introduction

Betaine is a safe and effective natural trimethylglycine found in microbes, animals, and plants. Since it is well-tolerated, inexpensive, and effective over a wide range of doses [1,2], betaine is an increasingly important supplement in animal husbandry. For example, betaine is an osmoprotectant [3,4]; it improves cysteine digestibility [5], antioxidant performance [6], and productivity [7,8]; and it regulates fat metabolism [9]. Therefore, betaine is beneficial for animal production. 

In animals, betaine regulates fat synthesis and metabolism via specific transport systems. Betaine prevents nonalcoholic fatty liver disease (NAFLD) induced by a high-fat diet in ApoE^−/−^ mice via the fibroblast growth factor 10/AMP-activated protein kinase signaling pathway [10]. In the swine industry, leanness and carcass quality are improved by the addition of 0.25% betaine [11]; moreover, betaine significantly reduces the cholesterol level of chicken breast meat [12]. Yusuf et al. [13] and Al-Sagan et al. [14] reported that blood triglyceride (TG) levels are increased by betaine supplementation in chickens. A systematic review and meta-analysis of randomized controlled trials suggested that dietary betaine supplementation reduces human body fat [15]. In addition, plasma betaine levels are inversely correlated with plasma TG levels in coronary artery disease patients [16]. Hence, betaine is an important factor for fat metabolism. 

Goose fatty liver (foie-gras) is a well-known delicacy and an unsaturated fatty acid-rich food composed of 50–60% lipids, which is produced using geese or ducks fed a diet rich in high-energy carbohydrates, where excess glucose is converted to fat via de novo lipogenesis in the liver, mainly in the form of TG [17,18,19,20]. The Landes goose is famous for production of foie-gras because it can store large amounts of fat in its liver [17,18,19,20].Interestingly, we found that betaine reduced sebum thickness and abdominal fat in Landes geese fed with high-energy carbohydrates, but increased liver weight due to fat accumulation [21]. Betaine also resulted in uniform lipid deposition, with a significant reduction in the number and volume of large lipid droplets [21]. This improves the economics of foie-gras production and animal welfare, but the exact mechanism is unclear. As a result, understanding the regulatory mechanisms underlying the physiological changes resulting from betaine supplementation in Landes goose liver could benefitfoie-gras production in the future. 

Although hepatic steatosis is induced in waterfowl by overfeeding with carbohydrates, unlike in other animals, liver steatosis is not characterized by hardening and necrosis [22]. Therefore, in this paper, we used high concentrations of glucose to induce fatty liver in primary goose hepatocytes, simulating overfeeding in vivo. The formation of fatty liver in geese or ducks is mainly a function of imbalanced fatty acid synthesis, transport, and β-oxidation in the liver, resulting in excessive lipid deposition [23,24]. We speculated that betaine may regulate fat metabolism in a specific manner not reported in other animals, which could improve the quality of foie-gras and decrease injury to geese resulting from overfeeding. However, other regulatory mechanisms may also protect geese from metabolic disorders, and investigation of these mechanisms could promote the efficiency of foie-gras production and animal welfare. We analyzed the effect of betaine on fatty acid β-oxidation and the size of lipid droplets. Furthermore, we investigated the expression of key genes in hepatic fat synthesis, secretion, and transport. Our results provide insight into the protective effects of betaine on Landes goose fatty liver and could provide a feasible and safe method to produce goose fatty liver. In addition, it provides mechanistic insight applicable to the treatment of fatty liver or obesity and other diseases related to fat metabolism.

## 2. Materials and Methods

### 2.1. Animals

A total of 34 healthy, hormone-free 3–4-week-old male Landes geese were purchased from the National Waterfowl Germplasm Resource Pool (Taizhou, Jiangsu, China). The geese were starved for 12 h before the experiment, with ad libitum access to drinking water.

### 2.2. Ethics Statement

The animal protocols were approved by the Institutional Animal Care and Use Committee of the Nanjing Agricultural University Animal Experiments Ethics Committee (NJAUIACUC201203001, Nanjing, China). Animal care and handling followed the IACUC guidelines.

### 2.3. Culture of Goose Primary Hepatocytes

The night before goose primary hepatocytes were cultured, rat-tail collagen was evenly spread on the plate bottom. The plate was tilted and subjected to UV irradiation overnight. Goose primary hepatocytes were isolated from Landes geese using a modified version of a two-step procedure [25]. Landes geese had fasted for 12 h and were intraperitoneally injected with heparin sodium (100 UI/kg body weight) and pentobarbital sodium (30 mg/kg body weight) for anesthesia. The geese were then perfused with 37 °C D-Hank’s washing solution at a rate of 25 mL/min until the liver turned pale yellow. Then, 0.18% trypsin solution was used for perfusion for approximately 6–7 min. The liver was separated carefully. We shredded the liver as finely as possible, and then separated the primary hepatocytes by 100, 200 filtering. The liver cell suspension was centrifuged at 600rpm for 6 min, repeated three times. Primary hepatocytes were diluted to 1 × 10^6^/mL and cultured in WME (Gibco, Grand Island, NY, USA) containing 100 IU/mL of penicillin, 100 mg/mL of streptomycin, 2 mM of glutamine (Sigma-Aldrich, Shanghai, China), and 10% fetal bovine serum (FBS; Wisent corporation, Nanjing, China). Primary hepatocytes were incubated at 37 °C in a humidified atmosphere containing 5% CO_2_.

### 2.4. Construction of Cell Model of Landes Goose Fatty Liver In Vitro

In order to simulate a Landes goose liver overfed with a high-carbohydrate diet that forms goose fatty liver, and to form a more typical fatty liver cell model to further study the effect of betaine on a Landes goose fat liver, we cultured the Landes goose primary hepatocytes with glucose of different concentrations (5, 10, 20, 30, 50, 100, 125, 150, 200 mmol/L). The Landes goose primary hepatocytes were isolated and cultured in the WME medium containing 10% serum for 24 h, and then in the serum-free medium for 24 h. After that, the cells were cultured for 48h in different concentrations of glucose with serum-free medium. The cell activity, TG concentration, and cell morphology changes of hepatocytes were detected.

### 2.5. 3-(4,5-Dimethylthiazol-2-yl)-2,5-diphenyltetrazolium Bromide (MTT) Assay

An MTT assay was performed to measure cell activity. Cells (~2.5 × 10^5^) were seeded into 96-well plates. The plates were coated with rat-tail collagen and sterilized by UV irradiation for 8–12 h. Hepatocytes were cultured in the medium containing 10% serum for 24 h, and then changed to the serum-free medium for 24 h. Each treatment was repeated three times for each plate. The cells were treated with serum-free medium containing different concentrations of glucose and incubated for 48 h. Control cells were cultured in serum-free medium (renewed every 24 h). We then removed the supernatant, and 20 µL of MTT stock solution and 80 µL of PBS were added to each well, and then incubated for 4 h at 37 °C. We then removed all the mediums and added 150 µL of DMSO to each well. We thoroughly mixed the plate and incubated it for 10 min at 37 °C. Cell activity was measured at 490 nm using a multimode reader (Biotek, Winooski, VT, USA). The cell activity curve was plotted with the average OD as the ordinate, and the glucose-induced groups with different concentrations as the abscissa.

### 2.6. TG Deposition Assay and Oil Red-O Staining

For the TG deposition assay, primary goose hepatocytes were seeded into 12-well plates at 3 × 10^5^/mL (nine replicates per concentration). The cells were fixed with 4% paraformaldehyde for 30 min, and equal amounts of Oil red-O solution were added to each well. The plate was incubated at room temperature for 30 min. Oil red-O solution was removed and then treated with 60% isopropyl alcohol for 20 s. Next, 60% isopropanol was added for 30 min to extract intracellular Oil red-O. Isopropanol containing Oil red-O was aspirated and the OD was measured. Regarding the gradient of the glucose concentration, 0 was used to represent the blank (detection wavelength 510 nm).

Goose primary hepatocytes were stained with Oil red-O to examine fat accumulation. Hepatocytes (5 × 10^5^/mL) were fixed in 4% paraformaldehyde for 30 min, rinsed with distilled water, and stained with Oil red-O for 30 min. The cells were then treated with 60% propylene glycol (*v/v*) for 20 s and rinsed in distilled water. The cells were examined under a phase-contrast microscope.

### 2.7. Betaine Effect on Landes Goose Fatty Liver Cells

The Langes goose fatty liver cells were co-cultured with different doses of betaine (0, 5, 10, 20, 30, 50, 100, 150, and 200 mmol/L) at the optimal inducing concentration of glucosein a serum-free medium. The cell activity, TG concentration, and changes in cell morphology were determined to reveal the effect of betaine on fatty liver cells of the Landes goose, and to screen the appropriate concentration of betaine to increase the content of triglyceride (TG). 

### 2.8. Determination of Mitochondrial Membrane Potential (MMP)

The MMP was determined using a JC-1 staining kit (Beyotime, Nanjing, China). Mitochondria were extracted from liver tissue and purified by differential centrifugation. Cells (1 × 10^5^) were washed twice in phosphate-buffered saline (2000 rpm, 5 min each) and suspended in 500 μL of JC-1 working solution. The cells were incubated in 5% CO_2_ at 37 °C for 20 min. After washing them twice with the incubation buffer, the cells were resuspended in 1 mL of incubation buffer and subjected to flow cytometry. The red-to-green fluorescence intensity ratio was analyzed using Cell Quest and ImageJ software (NIH). For detecting JC-1 monomers and polymers, the excitation wavelength was 490 and 525 nm, and the emission wavelength was 530 and 590 nm, respectively. The MMP changes were calculated by the relative ratio of red-to-green fluorescence.

### 2.9. Analysis of Lipid Droplets’ Distributionin Hepatocytes

The Oil red-O stain was used to detect the lipid droplets’ number and size after 24 h with different treatments. Ten representative Oil red-O images per group were randomly selected. The distribution and size of lipid droplets were analyzed by Image J. Abscissa stands for average intracellular lipid droplet size in square micrometers (μm^2^). The higher the value is, the larger the cross section of lipid droplets is. The 0 point represents no lipid deposition in cells. The ordinate represented the frequency of the highest point, and the higher the value, the more times that point occurs. 

### 2.10. RNA Isolation and Quantitative Reverse Transcription-Polymerase Chain Reaction (qRT-PCR)

Total RNA was isolated from cultured cells using the TRIzol reagent (Invitrogen, Shanghai, China) according to the manufacturer’s protocol. The RNA was reverse-transcribed into cDNA using the PrimeScript RT System Kit for Real-Time PCR (TaKaRa, Dalian, China). The mRNA levels were measured by qRT-PCR using SYBR^®^ Premix Ex Taq™ (TaKaRa, Dalian, China). Relative expression levels were calculated by the 2^−ΔΔCt^ method with β-actin as the endogenous control. The primers are listed in Table 1.

### 2.11. Data Analysis

Results were expressed as means ± SD. Statistical analysis was performed using SPSS (v.l6.0; SPSS Inc., Chicago, IL, USA). Comparison between groups was analyzed by one-way analysis of variance. Experiments involved three kinds of biological samples, each of which was run in triplicate. In this study, *p* < 0.05 means significant difference; *p* < 0.01 means extremely significant difference.

## 3. Results

### 3.1. Construction of the Cell Model of Landes Goose Fatty Liver In Vitro

**Figure 1:**
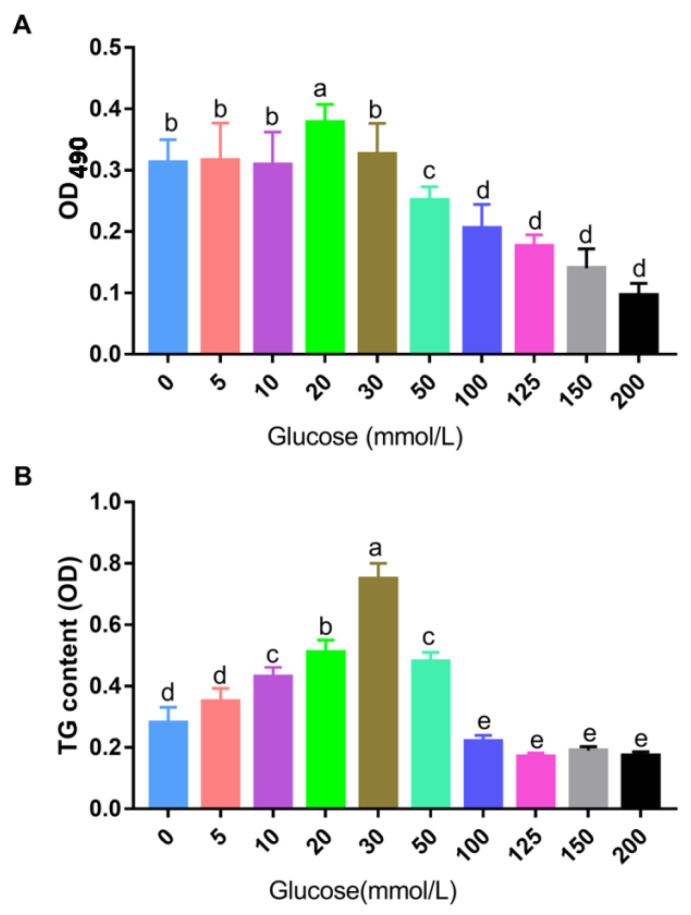
Effects of different concentrations of glucose on the activity and TG content of Landes goose primary hepatocyte. (**A**): Effect of glucose on Landes goose primary hepatocytes’ activity; (**B**): Intracellular TG content of Landes goose primary hepatocytes treated with glucose. Different lowercase letters in the bar indicate a significant difference in different groups (*p* < 0.05, *n* = 9).

In order to simulate Landes goose fatty liver feed with the high carbohydrate corn, we constructed the Landes goose fatty liver cell model only by adding different concentrations of glucose in vitro. Compared with the 0 mmol/L group, the activity of Landes goose primary hepatocytes was stable from 5 to 30 mmol/L of glucose at 48 h, and significantly enhanced when the glucose concentration was 20 mmol/L (*p* < 0.05). However, when the glucose concentration reached 100, 125, 150, and 200 mmol/L, the activity of goose hepatocytes decreased significantly *(p* < 0.05) (Figure 1A). The intracellular TG content is shown in Figure 1B, after 48 h of induction with different concentrations of glucose. The TG content was increased when the concentration of glucose was 5, 10, 20, and 30 mmol/L, but there was no significant difference between the concentration of the 5 mmol and 0 mmol/L groups; 50 mmol/L could promote the content of TG in cells, but that was significantly lower than that at 20 and 30 mmol/L (*p* < 0.05). When the glucose concentration increased to more than 100 mmol/L, the activity and content of TG in cells decreased sharply and significantly. This result showed that glucose can promote the content of TG within a certain concentration range, and the tolerance of liver cells to glucose declines when the glucose concentration is too high.

**Figure 2:**
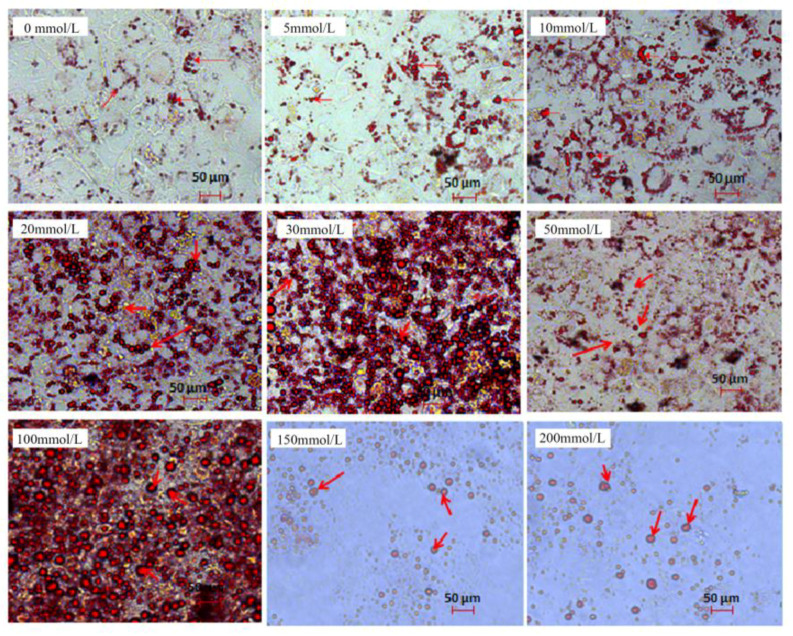
Lipid droplets were revealed by Oil red-O staining with different concentrations of glucose. Red arrows indicate lipid droplets.

Moreover, the Oil red-O staining indicated that the number of lipid droplets in Landes goose primary hepatocytes was significantly increased by glucose compared to the 0 mmol/L group, except 5 mmol/L (Figure 2). The lipid droplets in 20 and 30 mmol/L of glucose-induced groups were more intensive than other groups, which was consistent with the change trend of intracellular TG (Figure 1B). With 50 mmol/L of glucose, a large number of lipid droplets and cell fragments floated in the cell plate, and few cells survived when the glucose concentration was more than 100 mmol/L. These would explain why the glucose ≥50 mmol/L induced a sharp and significant decrease of the activity and the TG content of cells. In the range of cell tolerance, the lipid droplet deposition effect was obvious with the increase of glucose concentration. Interestingly, the big lipid droplets became more numerous and bigger than those in the control (CO) group, and the distribution of these lipid droplets became more and more uneven until the cells were broken. 

As comprehensive consideration of various experimental results, 20 mmol/L of glucose, with good cellular activity and moderate lipid droplet deposition effect, was selected as the optimal glucose concentration to construct the model of Landes goose fatty liver in vitro.

### 3.2. Effects of Betaine on TG Deposition in Landes Goose Fatty Liver Cells

**Figure 3:**
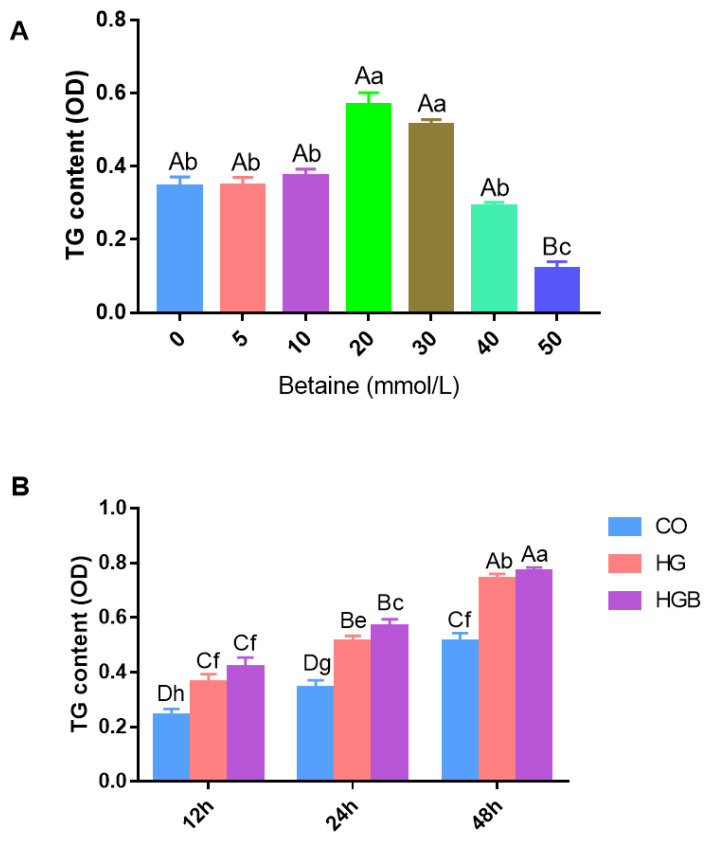
Effects of betaine on the intracellular TG content of Landes goose primary hepatocytes. (**A**): The TG content determent at 20 mmol/L of glucose with different doses of betaine; (**B**): The TG content at different times in different treatment groups. CO: control group (0 mmol/L of glucose + 0 mmol/L betaine); HG: 20 mmol/L of glucose + 0 mmol/L of betaine group; HGB: 20 mmol/L of glucose + 20 mmol/L of betaine combined treatment group. Different lowercase letters in the bar indicate a significant difference among different treatments (*p* < 0.05), and the different capitals indicate an extreme difference (*p* < 0.01), *n* = 9.

In order to determine the effect of betaine on TG content, Landes goose fatty liver cells were induced with 20 mmol/L of glucose and cultured with different doses of betaine at different times. As shown in Figure 3A, the TG content increased significantly with betaine at 20 and 30 mmol/L (*p* < 0.05). Consistent with this, Oil red-O staining also showed no difference with betaine at <10 mmol/L, however the density of lipid droplets was higher than other groups with betaine at 20 mmol/L (Supplementary Figure S1). The fat deposition had a downward trend with betaine >30 mmol/L, especially at 40 and 50 mmol/L (Supplementary Figure S1). In addition, there was no significant difference in cell activity in the betaine treatment groups (Supplementary Figure S2), so 20 mmol/Lof betaine treatment was used in this study. Compared to the high-glucose (HG) group, the TG content in the high-glucose and betaine-combined treatment (HGB) group was significantly increased at 24 h and 48 h (*p* < 0.05). Although the TG content in all groups increased significantly over time(12 h, 24 h, 48 h)(*p* < 0.01), the increase was greatest in the HGB group (Figure 3B).

### 3.3. Betaine Modulated Lipid Droplets Distribution and Size in Landes Goose Fatty Liver Cells

**Figure 4:**
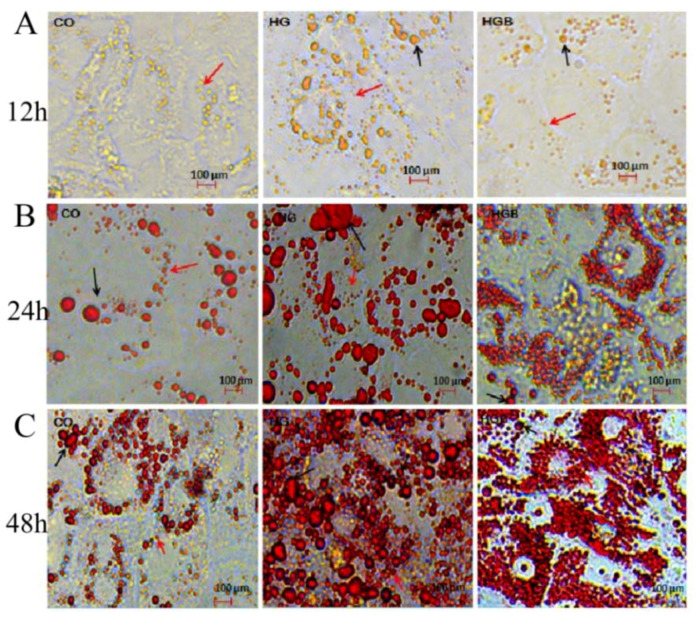
Oil red-O staining of Landes goose primary hepatocytes at different times and different treatments. Landes goose primary hepatocytes were starved in serum-free medium for 24 h and then cultured for different times ((**A**): 12 h, (**B**): 24 h, (**C**): 48 h with high glucose or high glucose and betaine. CO: control group (0 mmol/L of glucose + 0 mmol/L of betaine); HG: 20 mmol/L of glucose + 0 mmol/L of betaine group; HGB: 20 mmol/L of glucose + 20 mmol/L of betaine combined treatment group; red arrow: little droplets; black arrows: big droplets.

In the Oil red-O staining shown, when cultured for 12 h (Figure 4A), a few small lipid droplets could be seen in a few cells in the CO group. But there were great differences in the distribution of lipid droplets among cells in the HG group. Some cells had very different sizes of lipid droplet, some cells had a few small lipid droplets or even no lipid droplets. However, in the HGB group, there were a number of evenly distributed small lipid droplets and few large lipid droplets in most cells. At 24 h (Figure 4B), the lipid droplets were more than 12 h in all groups, and different sizes were detected in the CO group. The distribution and size of lipid droplets in the HG group were uneven and the number of large lipid droplets was the largest, and each large lipid droplet volume accounted for more than half of the cell. In the HGB group, the number of lipid droplets was the most, but the size was the smallest, and they were evenly distributed around the cell membrane. At 48 h (Figure 4C), there were a large number of lipid droplets in each group, and the differences in the number and distribution between large and small lipid droplets among groups were more obvious; even many large lipid droplets were found in the CO group. More and more uneven lipid droplets were found in the HG group, and the size difference of lipid droplets was larger. The distribution of small lipid droplets in the HGB group was more uniform than that in 24 h. In addition, many small lipid droplets were deposited in almost every cell.

**Figure 5:**
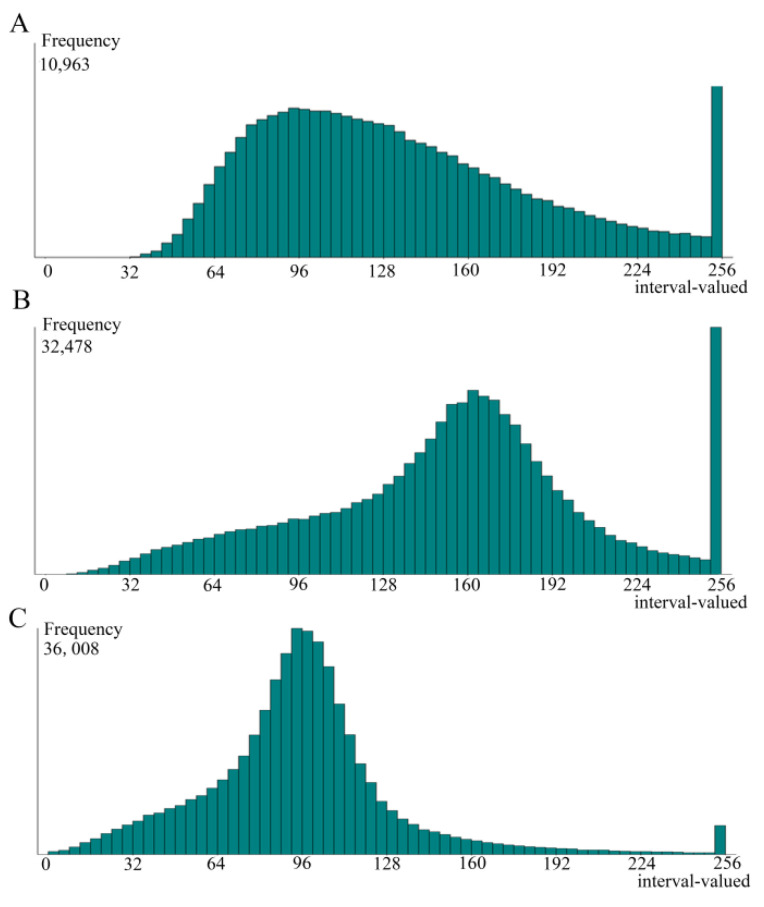
The statistics of lipid droplets size and distribution of different groups. Abscissa stands for average intracellular lipid droplet size in square micrometers (μm^2^). The ordinate represented the frequency of the highest point, and the higher the value, the more times that point occurs. (**A**): control group (0 mmol/L of glucose + 0 mmol/L of betaine); (**B**): 20 mmol/L of glucose + 0 mmol/L of betaine group; (**C**): 20 mmol/L of glucose + 20 mmol/L of betaine combined treatment group.

In order to further compare the distribution and size of lipid droplets in fatty liver cells among different groups, ten representative images per group were randomly selected at 24 h. The lipid droplets were counted using Image J software. In the CO group (Figure 5A), the size of lipid droplets concentrated in the interval of 64–160 μm^2^ and the overall distribution trend was mild. Moreover, the medium size lipid droplets were predominately in the CO group, and there were a certain number of cells without lipid deposition, and the large lipid droplets in cells were not uniform. In the HG group (Figure 5B), the lipid droplets also fell in the range of 256 μm^2^. In addition, the lipid droplets were concentrated in the interval of 128–192 μm^2^ and the overall distribution trend was steep; however, the sides were mild. That indicated large lipid droplets predominated in the HG group, and small fat droplets accounted for a very small percentage. Meanwhile, the distribution between large and small lipid droplets was uneven. In the HGB group (Figure 5C), the majority of lipid droplets fell at around 96 μm^2^. Moreover, it was a variation from the interval of 0 to 256 μm^2^ and the number of lipid droplets was fewer than 160 μm^2^. That indicated betaine treatment could promote small lipid droplets deposition, not large lipid droplets, and small lipid droplets were evenly distributed.

### 3.4. Betaine Restored Fatty Acid β-Oxidation and Promoted Fat Oxidative Decomposition

**Figure 6:**
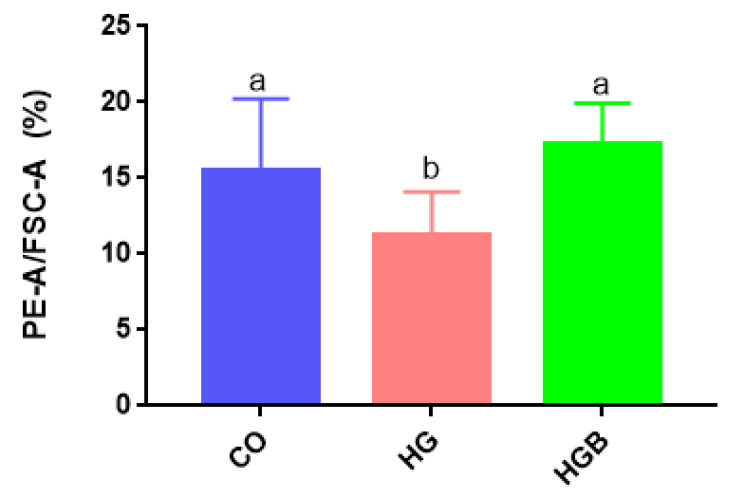
The changes of mitochondria membrane potentials in Landes goose primary hepatocytes in different treatment groups. After 24 h starved in serum-free medium, Landes goose primary hepatocytes were cultured for 48 h with high glucose or high glucose and betaine treatment. Different lowercase letters indicate a significant difference (*p* < 0.05). CO: control group (0 mmol/L of glucose + 0 mmol/L of betaine); HG: 20 mmol/L of glucose + 0 mmol/L of betaine group; HGB: 20 mmol/L of glucose + 20 mmol/L of betaine combined treatment group.

Fatty acid β-oxidation in mitochondria enhances the activity of fat decomposition enzymes, thus reducing fat deposition. The β-oxidation typically takes place in mitochondria and is suppressed by impaired mitochondrial function. Changes in MMP could reflect mitochondrial function and thus, indirectly, changes in intracellular fatty acid β-oxidation. The MMP of the HG group was significantly lower than that of the CO and HGB groups (*p* < 0.05) (Figure 6). Compared to the CO group, the MMP of the HGB group was not significantly different (*p* > 0.05). As a result, betaine could protect the cell MMP, and promote the β-oxidation of fat compared with the HG group. 

### 3.5. Betaine Inhibited the Expression of Key Genes in Fat Synthesis, Deposition, and Metabolism

**Figure 7:**
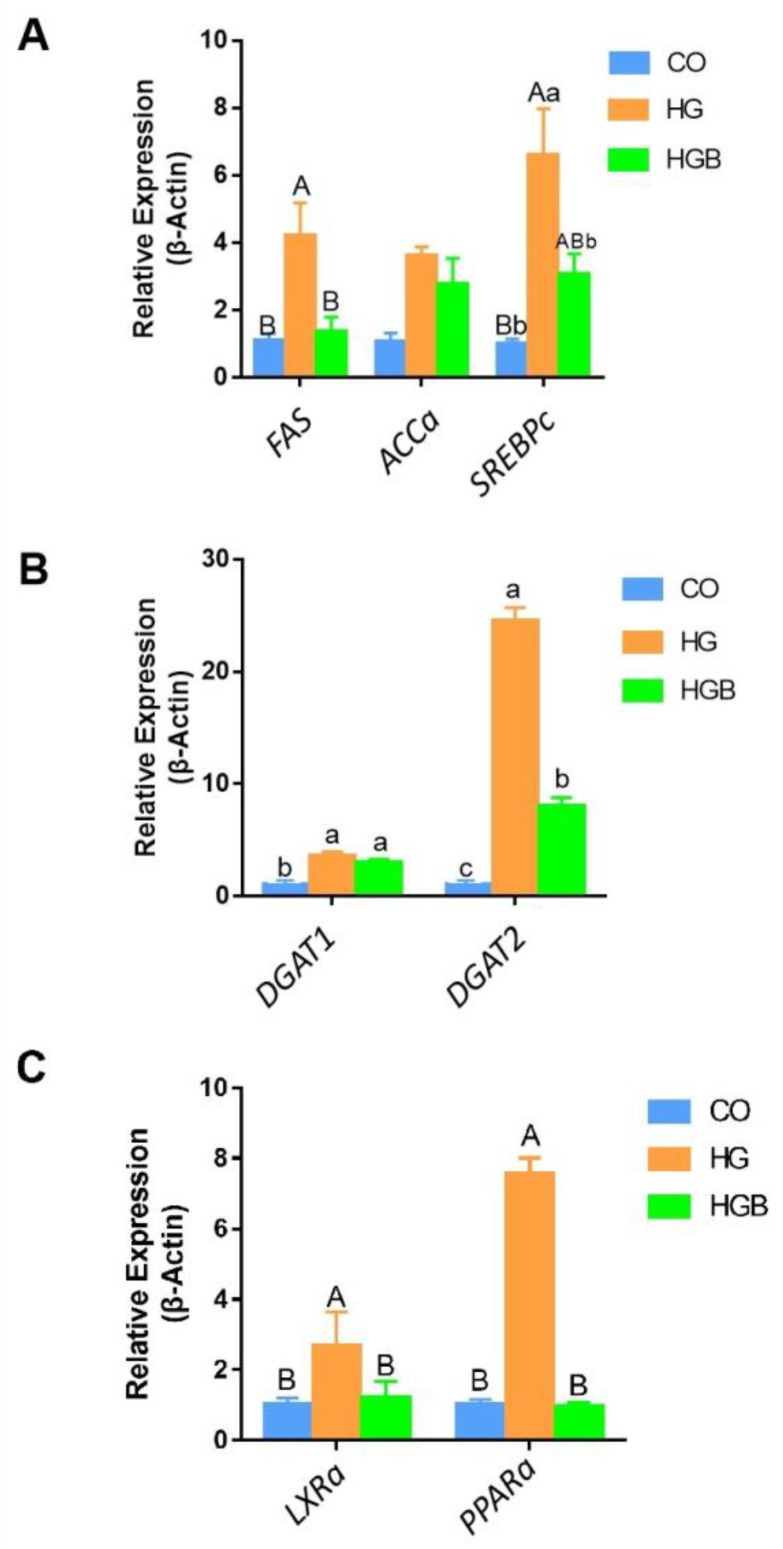
The changes of key genes for fat synthesis, deposition, and metabolism with different treatments. (**A**): The key genes’ (*FAS*, *AACα*, and *SREBPc*) expression levels of fat synthesis pathway; (**B**): The key genes’ (*DGAT1* and *DGAT2*) expression levels of fat deposition; (**C**): The key genes’ (*LRXα* and *PPARα*) expression levels of lipid regulation metabolism. Different lowercase letters indicate a significant difference (*p* < 0.05), and the different capitals indicate an extreme difference (*p* < 0.01).CO: control group (0 mmol/L of glucose + 0 mmol/L of betaine); HG: 20 mmol/L of glucose + 0 mmol/L of betaine group; HGB: 20 mmol/L of glucose + 20 mmol/L of betaine combined treatment group.

The expression levels of fatty acid, sterol, regulatory, element-binding proteins (*SREBPc*) and fat synthetase (*FAS*) in the HGB group were significantly lower than that in the HG group, whereas acetyl-CoA carboxylase (*ACCa*) showed no significant difference among the groups (Figure 7A).That indicated that betaine inhibited the synthesis of fatty acids. The expression level of diacylglycerol acyltransferase 1 (*DGAT1*), a key gene in fat deposition, was not significantly different between the HG and HGB groups (*p* > 0.05), but was higher in both than the CO group (*p* < 0.05). The expression level of diacylglycerol acyltransferase 2 (*DGAT2*) in the HGB group (Figure 7B) was lower than the HG group, but higher than the CO group (*p* < 0.05). The expression of liver X Receptor α (*LXRα*) and the peroxisome proliferator-activated receptor (*PPARα*), key genes in intrahepatic lipid metabolism, showed a similar change trend. The expression of both genes increased in the HG group and decreased in the HGB group, which is relative to the level of the CO group (*p* < 0.01, Figure 7C). These results indicated that betaine may inhibit the expression of key genes at synthesis, deposition, and metabolism in Landes goose fatty liver cells.

### 3.6. Betaine Promoted the Expression of Adipolytic and Transport-Related Genes

**Figure 8:**
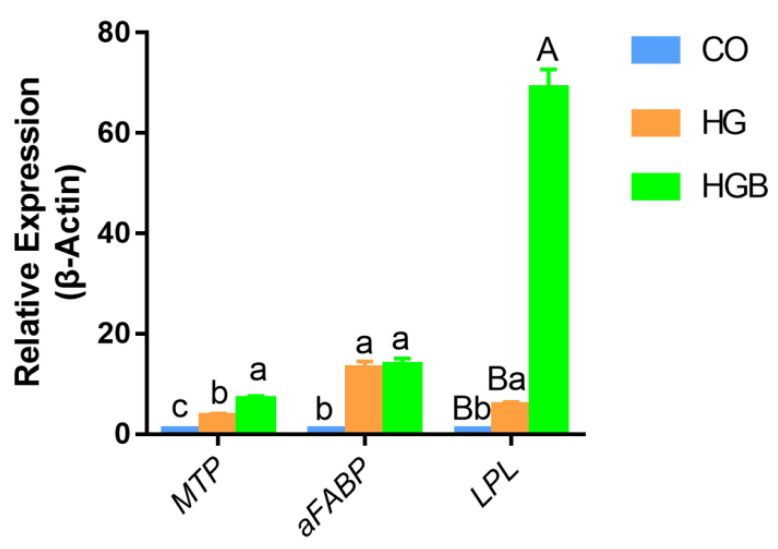
The relative mRNA expression levels of key genes (*MTP*, *aFABP*, and *LPL*) in fatty acids transportation and decomposition pathway with different treatments. Different lowercase letters indicate a significant difference (*p* < 0.05), and the different capitals indicate an extreme difference (*p* < 0.01). CO: control group (0 mmol/L of glucose + 0 mmol/L of betaine); HG: 20 mmol/L of glucose + 0 mmol/L of betaine group; HGB: 20 mmol/L of glucose + 20 mmol/L of betaine combined treatment group.

Adipocyte fatty acid-binding protein (*aFABP*), microsomal triglyceride transfer protein (*MTP*), and lipoprotein lipase (*LPL*) are key genes in fatty acid decomposition and transport, and showed similar variations in expression. Their expression levels were lowest in the CO group, highest in the HGB group, and intermediate in the HG group (Figure 8). Compared to the HGB group, the expression level of *aFABP* was not significantly different (*p* > 0.05), unlike that of *MTP* (*p* < 0.05); *LPL* expression was significantly different (*p* < 0.01) in the HG group. Therefore, betaine promotes the transport, but the adipolysis effect is not obvious.

## 4. Discussion

Goose fatty liver (foie-gras) mainly results from the huge storage of newly synthesized TG in the liver from dietary carbohydrates in response to overfeeding. Waterfowl (including geese and ducks) are used for production of foie-gras via overfeeding with a high-energy diet rich in carbohydrates. Unlike mammals, waterfowl show considerable fat production in the liver. Indeed, our results show that TG deposition in the CO group at 48 h was similar to the level reached by the HG group at 12 h, indicating that primary hepatocytes of the Landes goose have a high propensity for lipogenesis, which may explain the higher fat deposition in the Landes goose liver than in the livers of other common goose breeds. This also indicates that it should be feasible to construct a fatty liver model in vitro using goose hepatocytes with a high-glucose medium. However, overfeeding exerts adverse effects on hepatocyte function and physiology [26,27]. Liver damage caused by mechanical or artificial overfeeding needs to be ameliorated by improving feeding methods to promote animal welfare. 

Betaine regulates fat metabolism, but the regulatory effect varies among species [28,29,30,31]. In humans, betaine prevents alcohol-induced steatosis by reducing liver fat deposition [32]; meanwhile, increased NAFLD severity is associated with betaine insufficiency, and elevated betaine levels improve liver injury and reduce fat deposition [33].In livestock and poultry, betaine increases carcass quality by increasing intramuscular fat and improves the egg-laying rate by inducing hepatic lipogenesis. However, in the Landes goose, betaine reduces abdominal sebum fat thickness while increasing liver weight [21], an effect that could benefit the production of goose fatty livers. In this study, betaine supplementation increased TG content in Landes goose fatty liver cells while improving cellular activity, resulting in a healthier steatosis phenotype. Specifically, betaine inhibited the formation of large lipid droplets, resulting in more evenly distributed lipid droplets, which reduced liver damage while increasing fat deposition. By contrast, the low cell activity and marked steatosis in the HG group may have been caused by large lipid droplets that resulted in cell lysis. Moreover, lipid droplets in the HG group were distributed unevenly: some cells contained many lipid droplets, whereas others had none. Therefore, betaine appears to result in even intracellular lipid droplet deposition, reduced damage to hepatocytes, and a healthier steatosis phenotype in fatty liver cells. This is consistent with our previous studies, where in Landes geese were not artificially or mechanically overfed with the high-energy food with ad libitum intake [28]. In this paper, we also found that betaine not only increased the TG content, but also regulated the size of lipid droplets. If lipid droplets become too big and uneven, cell rupture will occur, which is detrimental to goose health. We hope to improve the production and quality of foie-gras by supplementing with betaine.

Hepatic steatosis results from imbalances in hepatic lipid metabolism leading to excessive lipid deposition. Ipsen et al. reported that hepatic steatosis results from elevated fatty acid uptake and synthesis and suppressed fatty acid oxidation [34]. Mitochondrial function plays an important role in this process. Liver cells are rich in mitochondria, and mitochondrial dysfunction is a marker of NAFLD [35,36]; betaine relieves endoplasmic reticulum stress and restores mitochondrial function [1]. In this study, the MMP was low in the HG group and significantly higher in the HGB group, indicating betaine may influence fatty acid β-oxidization. Previous studies suggest betaine may affect fat composition and deposition in a species-specific manner: Hu et al. showed that in newly hatched chicks, betaine induces cholesterol accumulation in the liver [37]. However, in human HepG2 cells, betaine inhibits fat accumulation and increases mitochondrial content and activity [30]. In this study, betaine restored the MMP, thereby promoting fat turnover via hydrolysis and oxidation, which in turn inhibited intrahepatic fat synthesis.

Betaine increased the TG content in the liver cells of Landes geese, but significantly decreased the expression of genes related to fat synthesis (*SREBPc*, *FAS*, and *ACCa*) and metabolism (*LXRα* and *PPARα*). These results are consistent with a previous report that betaine inhibits fatty acid synthesis; the increased cellular TG content may be caused by specific properties of Landes goose liver cells. Betaine significantly increased the expression of key genes in the lipid transport pathway. LPL is involved in the plasma lipid in tissues and is positively correlated with goose or duck fatty liver weight; however, hepatic LPL is largely absent in other adult mammals [38]. Liver LPL is important in fatty acid hydrolysis and transport, which is required for TG hydrolysis in peripheral liver tissue and release of fatty acids into liver. Generally speaking, the expression of LPL is eliminated in adult animals and is only re-expressed under certain situations; for example, morbidly obese individuals have higher LPL synthesis in the liver. LPL enables the liver to hydrolyze TG from chylomicrons and VLDL, not only helping to clear plasma lipids, but also leading to an increase in hepatic uptake of NEFA and, therefore, steatosis [39]. It is reported that LPL is a key enzyme in the regulation of adipose tissue deposition in poultry and that increases in its activity facilitate fat deposition. Consistent with prior reports [40,41,42], betaine increased the *LPL* mRNA level almost ten-fold compared with the HG group. Therefore, we speculate that the different expression patterns of *LPL* in liver cells may be the main reason for the different effects of betaine on characteristics of adipose deposition and hepatic steatosis between waterfowl and mammals, since increased hepatic LPL expression in waterfowl may divert TG away from storage in adipocytes and facilitate storage in the liver.

In this study, betaine increased TG content and changed the size and distribution of droplets in liver cells, which has not been reported previously. Moreover, different concentrations of betaine had significantly different effects on lipid deposition in liver cells. Whereas betaine supplemented at <10 mmol/L had no effect on cellular TG content; the optimum betaine at concentrations of 20–30 mmol/L increased TG content, and betaine concentrations of >30 mmol/L decreased TG content. This may be due to a balance between the lipid storage capacity of Landes goose hepatocytes and the lipid-lowering effect of betaine. The expression of the fat transport gene *MTP* was increased more than two-fold by betaine. MTP is present in cytosolic lipid droplets of hepatocytes [43] and is particularly abundant in small lipid droplets [44]. Swift et al. showed that MTP expression is negatively correlated with lipid droplets’ size in mice, suggesting that MTP plays a role in determining size of lipid droplet [45]. Liver lipid droplet size was decreased by betaine; therefore, we speculate that this effect could be mediated by changes in the regulation of *MTP*. Therefore, betaine supplementation in the Landes goose, which decreases sebum thickness and abdominal fat, and increases liver weight [21], may result from an imbalance of multiple fatty acid metabolic pathways. Although fatty acid synthesis within the liver is down-regulated, and fatty acid mobilization, oxidation, and secretion are up-regulated, peripheral TG hydrolysis and uptake of circulating fatty acids into the liver appears to outweigh these effects. This results in a net reduction in peripheral fat (i.e., sebum thickness) and an increase in liver TG content and turnover, providing confirmation of and mechanistic insight into the results previously observed *in vivo.*

## 5. Conclusions

The Landes goose is a famous breed commonly used for foie-gras production, whose primary hepatocytes facilitate high levels of fat deposition. Here, an in vitro model of fatty liver was successfully established by exposing primary cells to a high-glucose medium. In betaine-treated cells, TG content increased, but steatosis was characterized by a healthier phenotype. Betaine improved fat deposition by regulating genes involved in fat synthesis, secretion, and transport; restoring the MMP; and preventing an increase in lipid droplet size; and by promoting the even distribution of lipid droplets. These results provide insight into how betaine supplementation may be leveraged to facilitate the production of high-quality foie-gras and promote animal welfare.

## Figures and Tables

**Table 1:** Parameters of oligo-nucleotide primer pairs for the genes.

GenBank No.	Target Genes	Anneal. T (℃)	Primer Sequence	Product
M26111	*β-actin*	65	F: 5′-ACCACCGGTATTGTTATGGACT-3′ R: 5′-TTGAAGGTGGTCTCGTGGAT-3′	398 bp
J03541	*ACCα*	59	F: 5′-TGTGGCTGATGTGAGCTTTC-3′ R: 5′-ACTGTCGGGTCACCTTCAAC-3′	152 bp
NM205155	*FAS*	59	F: 5′-AATCCATGGCTAAACGCATC-3′ R: 5′-GGCCATTTACTTCCTGTGGA-3′	216 bp
AF481797	*PPARα*	50	F: 5′-AGACCCTTGTGGCAAAACTG-3′ R: 5′-TAGGCTACCAGCATCCCATC -3′	247 bp
AF432506	*aFABP*	60	F: 5′-CAGCATCAATGGTGATGTGA-3′ R: 5′-TCTCTTTGCCATCCCACTT-3′	179 bp
AF492498	*LXRα*	57	F: AGCAGGTCTGCAGTTCGAGT R: GGCTTCCACATAGGTGTGCT	181 bp
AJ310768	*SREBPc*	66	F: 5′-GCGCTACCGCTCATCCATCA-3′ R: 5′-GGTCGGCATCTCCATCACCT-3′	283 bp
GW342946	*DGAT1*	60	F: 5′-CCTGAGGAACTTGGACACG-3′ R: 5′-CAGGGACTGGTGGAACTCG-3′	265 bp
GW342947	*DGAT2*	60	F: 5′-CGCCATCATCATCGTGGT-3′ R: 5′-CGTGCCGTAGAGCCAGTTT-3′	113 bp
XM_013199906	*MTP*	60	F: 5′-CCCGATGAAGGAGAGGAA-3′ R: 5′-AAAATGTAACTGGCCTGAGT-3′	85 bp
XM_013188252	*LPL*	55	F: 5′-TGCCCTCACACGCCTCTC-3′ R: 5′-TCTGAATGCCGATGCTGC-3′	95 bp

## Data Availability

The original data in the article can be obtained directly from the corresponding author. The data are not publicly available due to privacy restrictions.

## Supplementary Materials

The following supporting information can be downloaded at: https://www.mdpi.com/article/10.3390/ani12121530/s1. Figure S1: Effects of different concentrations of betaine on Landes goose primary hepatocyte activity. Goose primary hepatocytes were cultured with different concentrations of betaine for 48 h and stained by Oil red-O; Figure S2: Effects of different concentrations of betaine on Landes goose primary hepatocyte activity. Betaine at <30 mmol/L were used to culture the goose primary hepatocyte and the cell activity was detected by MTT at 12, 24, 36, 48, 60, and 72 h.
