# Supplementary material for: Betaine Promotes Fat Accumulation and Reduces Injury in Landes Goose Hepatocytes by Regulating Multiple Lipid Metabolism Pathways
Source: Animals (Basel). 2022 Jun 13;12(12):1530. doi: 10.3390/ani12121530 (PMC9219492; doi:10.3390/ani12121530)
Supplement: Supplementary file 1 [file animals-12-01530-s001.zip › animals-1763869-supplementary.pdf]

Supplementary materials

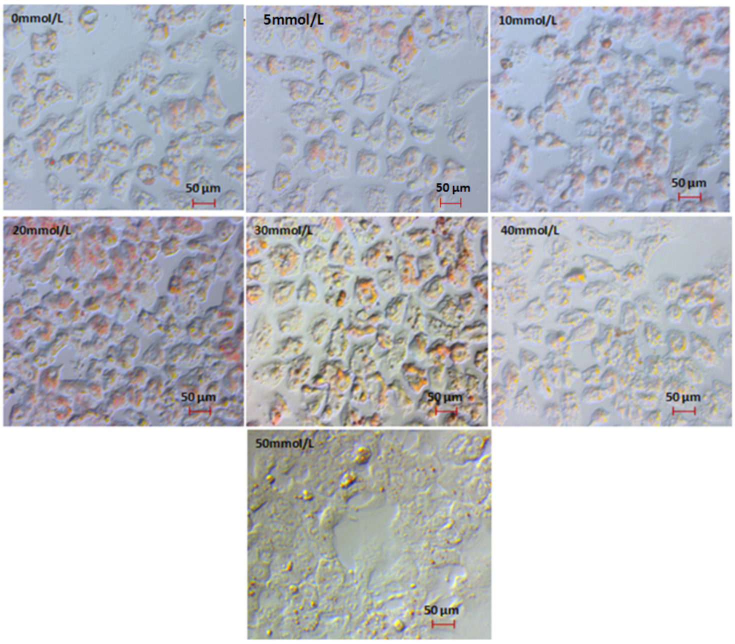

Supplementary Figure S1. Effects of different concentrations of betaine on Landes goose primary hepatocyte activity. Goose primary hepatocytes were cultured with different concentrations of betaine for 48 h and stained by Oil red-O.

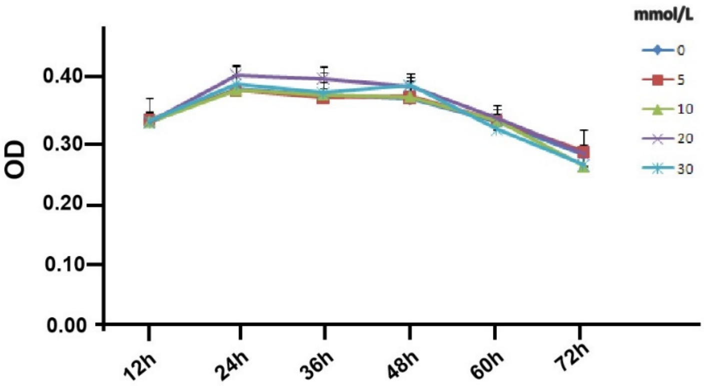

Supplementary Figure S2. Effects of different concentrations of betaine on Landes goose primary hepatocyte activity. Betaine at < 30 mmol/L were used to culture the goose primary hepatocyte and the cell activity was detected by MTT at 12, 24, 36, 48, 60, and 72 h.
